# Effects of Activating Mutations on EGFR Cellular Protein Turnover and Amino Acid Recycling Determined Using SILAC Mass Spectrometry

**DOI:** 10.1155/2015/798936

**Published:** 2015-11-24

**Authors:** Michael J. Greig, Sherry Niessen, Scott L. Weinrich, Jun Li Feng, Manli Shi, Ted O. Johnson

**Affiliations:** ^1^Worldwide Medicinal Chemistry, Pfizer, Inc., La Jolla Laboratories, 10770 Science Center Drive, San Diego, CA 92121, USA; ^2^Oncology Research Unit, Pfizer, Inc., La Jolla Laboratories, 10770 Science Center Drive, San Diego, CA 92121, USA

## Abstract

Rapid mutations of proteins that are targeted in cancer therapy often lead to drug resistance. Often, the mutation directly affects a drug's binding site, effectively blocking binding of the drug, but these mutations can have other effects such as changing the protein turnover half-life. Utilizing SILAC MS, we measured the cellular turnover rates of an important non-small cell lung cancer target, epidermal growth factor receptor (EGFR). Wild-type (WT) EGFR, EGFR with a single activating mutant (Del 746–750 or L858R), and the drug-resistant double mutant (L858R/T790M) EGFR were analyzed. In non-small cell lung cancer cell lines, EGFR turnover rates ranged from 28 hours in A431 cells (WT) to 7.5 hours in the PC-9 cells (Del 746–750 mutant). The measurement of EGFR turnover rate in PC-9 cells dosed with irreversible inhibitors has additional complexity due to inhibitor effects on cell viability and results were reported as a range. Finally, essential amino acid recycling (K and R) was measured in different cell lines. The recycling was different in each cell line, but the overall inclusion of the effect of amino acid recycling on calculating EGFR turnover rates resulted in a 10–20% reduction in rates.

## 1. Introduction

Epidermal growth factor receptor (EGFR) is a transmembrane cell-surface receptor from the epidermal growth factor family. EGFR is a prominent target in cancer therapy—overexpression of EGFR was first identified in malignant gliomas in 1987 [1]. About 20% of patients with non-small cell lung cancer (NSCLC) have tumor associated EGFR mutations. These mutations fall into two mutually exclusive classes which together represent 90% of all observed EGFR mutations. The mutations are either an in-frame deletion in exon 19 (Del19) or a single point mutation where leucine 858 is converted to an arginine (L858R). Both somatic mutations result in ligand independent activation of EGFR and its effector pathways driving cellular proliferation and survival. Patients with these EGFR mutations are highly responsive to first-generation ATP competitive inhibitors such as erlotinib and gefitinib. These tyrosine kinase inhibitors (TKIs) target the EGFR tyrosine kinase domain by reversibly binding to the ATP-binding site. Unfortunately, within about 12 months, most patients acquire resistance to these drugs. In about half of these cases the resistance was a result of a secondary mutation in EGFR where the gatekeeper threonine was converted to a methionine (T790M). First-generation inhibitors such as erlotinib are ineffective in inhibiting the activity of both predominant double mutant (DM) forms of EGFR. This has led to the development of third-generation EGFR irreversible inhibitors aimed at improving the duration of response when targeting these mutants.

While the majority of marketed small molecule drugs are reversible binders, covalent inhibitors make up approximately one-third of the marketed enzyme inhibitors. These include widely used drugs such as aspirin, penicillin, clopidogrel (an antiplatelet agent), and many proton pump inhibitors. Currently there are several covalent inhibitors approved or in the clinic targeting EGFR or its mutants. Afatinib and dacomitinib are second-generation covalent inhibitors targeting WT and single mutant EGFR for NSCLC and AZD9291 and CO-1686 are third-generation covalent inhibitors in the clinic targeting the DM forms of EGFR [2–5]. The rationale to develop an irreversible inhibitor may include presence of a highly accessible reactive site on the target (i.e., a cysteine residue) which may enhance selectivity or achieving a prolonged pharmacodynamic effect if the target protein has a slow turnover rate. Potential risks of such drugs include long term toxicities and side effects due to nonspecific or off-target binding and immune response to the protein-drug adduct. These risks are often offset by the potential for a lower dose and longer term inhibition afforded by irreversible binders. Competition with high concentrations of natural substrates, such as ATP, can also be negated by covalent binding. The duration of inhibition of the target protein is directly related to its turnover rate, the pharmacodynamic, and to some extent the pharmacokinetic properties of the compound. As such we measured the turnover rate of wild-type and mutant EGFR.

Protein turnover in the cell can range from minutes to days. Protein turnover, as generally calculated, encompasses degradation and synthesis rates and is measured at the point the curves intersect. The principal protein degradation pathway is the ubiquitin-proteasome pathway which takes place in the cytoplasm and nucleus. The other main catabolic protein pathway is the autophagy-lysosomal system. A third factor that affects protein turnover rate is amino acid recycling. Unlike excess glucose that can be stored long term in our bodies as glycogen, there is no long term storage mechanism for excess amino acids. They are either rapidly used in new protein synthesis or metabolized to acetyl-CoA, urea, or components of the citric acid cycle. To further complicate this, rates of recycling may also be different for essential and nonessential amino acids. It has been suggested that protein degradation remains constant, while protein synthesis is variable depending upon external perturbations [6].

The turnover rates of mammalian cellular proteins were measured using isotopically labeled amino acids as early as 1959 [7]. Subsequently, radiolabeled (typically [^35^S] methionine) pulse-chase or washout experiments were typically used to estimate protein half-lives. These assays have inherent drawbacks including the requirement for fluorescent or radioactive tags and/or antibodies for detection. In the case of using antibodies for identification of bands to measure on a gel (radiolabeled or fluorescent), the method is at the mercy of the specificity of the antibody. In order to address these concerns, Pratt and colleagues used stable isotope labeling by amino acids in cell culture (SILAC) with mass spectrometry (MS) to measure protein turnover rates [8, 9].

There are several advantages of using SILAC MS to measure protein turnover rates. First, the actual turnover rate is measured, not just either a degradation or synthesis rate, as is measured using other techniques. SILAC labeling allows the measurement of the loss of unlabeled peptides (degradation and cell doubling dilution) as well as the synthesis of new protein via labeled peptides; thus we take into account both processes. The half-life (or decay rate) is the number we assign the point at which 50% of the “old” proteins have been replaced by new protein. Final turnover rate *t*_1/2_ is the protein turnover rate adjusted for cell dilution effects. Compounds that inhibit translation such as cycloheximide are not necessary, eliminating uncertainty caused by the potential disruption of other cellular processes by these chemicals. Specifically for EGFR, cycloheximide induces ligand independent internalization of EGFR within 10 minutes, which will not only affect the activity of the receptor, but also most likely affect the turnover rate [10]. Mass spectrometry (MS) studies have shown that 10% of proteins in HeLa cells had half-lives less than six hours, indicating rapid turnover, while another 10% had half-lives greater than thirty hours [11]. Both of these extremes can have dramatic effects on the pharmacodynamic properties of irreversible inhibitors.

Several factors can alter protein synthesis, degradation, and overall turnover rates. Phosphorylation can target a protein for degradation and thereby increase its turnover rate. This effect has been observed with the phosphorylation of ER*α* and GADD34 [12, 13]. Conversely, phosphorylation of a highly conserved serine residue on transcription factor ΔFosB protects it from proteasomal degradation [14]. Other proteins have been reported to have a faster turnover when activated, including the activation of receptor tyrosine kinases by growth factors and insulin receptors by insulin [15, 16]. EGFR in wild-type cells was found to have a half-life of about 20 hours, but when EGF was added to the cells, the half-life dropped to about 9 hours [17]. EGFR degradation is found to primarily happen when the protein is ubiquitinated after ligand-dependent activation and phosphorylation. The protein is internalized and sent to endosomes to be marked for degradation. Interestingly, internalization alone of EGFR does not result in degradation via lysosomes and could potentially delay degradation of the protein by keeping it from being activated [18].

Amino acid recycling is an important factor to consider when calculating protein turnover rates. Over multiple days, it was found in the plant* Lemna minor* that 29–50% of the measured amino acids were recycled [19]. More recently, in a SILAC study of protein turnover in HeLa cells, 10–20% of the peptides observed contained recycled amino acids at 48 hours [11]. Another study concluded that the preferred amino acid supply for protein synthesis is from the extracellular pool and not amino acid recycling [20]. Due to missed cleavages during the enzymatic digestion step, the incorporation of both labeled (new) amino acids and unlabeled (recycled) amino acids in the same peptide can be measured [11, 20]. The presence of both types of amino acids in one peptide only occurs when unlabeled amino acids were recycled, providing a method to observe amino acid recycling. Turnover rates measured by only accounting for peptides with completely heavy and completely light lysine (K) and arginine (R) underestimate the actual turnover rate. Depending on the propensity for the cell to recycle amino acids, the turnover rate could be much faster than previously thought. We used isotopically labeled lysine and arginine for our SILAC experiments. Lysine is an essential amino acid and arginine is a semiessential amino acid; therefore it would be expected that the recycling of these amino acids would be more than nonessential amino acids. Thus the impact on protein turnover rate may be dependent on which amino acids are labeled.

The A431 cell line is a human epithelial carcinoma cell line that overexpresses EGFR and is a widely used cell line for studying the mitogen-activated protein kinase (MAPK) pathway. Data from this cell line was used to benchmark WT EGFR, which could be used as a comparator for protein turnover rates in mutant EGFR cell lines.

Protein degradation rates can be used to estimate protein turnover, although using degradation rates alone most likely results in an overestimation of the turnover time. EGFR degradation in A431 cells was previously measured by Stoscheck and Carpenter, who found the rate of EGFR degradation in A431 cells to have *t*_1/2_ = 20 hrs. This rate was determined by labeling the receptor* in vitro* with radioactive amino acid precursors and chased with nonradioactive media. Other measurements of EGFR half-lives in the absence of EGF range from 10 to 48 hours [21–23].

In additional experiments by Stoscheck and Carpenter in A431 cells, they analyzed degradation rates of EGFR when EGF was added at the beginning of the chase and the half-life of EGFR decreased to 8.9 hrs [17]. The half-life of EGFR in the presence of EGF was also measured in HB2 and 184A1 cells (human mammary epithelial cells) and found to be reduced from 14 and 11 hours to 1.6 and 2 hours, respectively [21]. The half-life of activated EGFR was also measured in 786-0 clear cell renal cell carcinoma cells and found to be 1.5–4 hours depending on the presence of von Hippel-Lindau protein or hypoxia [24].

Protein turnover rates can also be affected by specific inhibitors. It is known that the addition of kinase inhibitors can affect the turnover rate of the target protein; for example, the addition of AZD1152-HQPA, a potent Aurora kinase B inhibitor, increased the turnover rate of Aurora B via the ubiquitination and proteasomal degradation pathway [25]. Another study investigating the effects of drug-mediated inhibition of the Hsp90 molecular chaperone on the global proteome found changes in the synthesis and degradation rates of many proteins [26].

## 2. Materials and Methods

In our experiments, we measured the ratio of the abundance metabolically “light” or “heavy” peptides through the incorporation of heavy arginine [^13^C_6_, ^15^N_4_] and heavy lysine [^13^C_6_, ^15^N_2_]. From this data, we plotted a one-phase exponential decay curve to determine turnover half-life. We did not add a calculation to account for amino acid recycling, but we did investigate amino acid recycling in several cell lines. The amino acid recycling effect on turnover was not added to the half-life equation because many variables, such as time to reach steady state amino acid pool, could not be readily calculated.

For each time point in the following experiments, two or three biological replicates were analyzed as well as two or three technical replicates. For the H3255 cells at the 24-hour time point, the same sample was run thirteen times with a H/L ratio of 0.14 and a standard deviation of 0.0049. This accuracy was observed consistently; thus there was a reduction of technical replicates for later analyses. Time point zero for each set of cells was initially measured 3–5 times and consistent incorporation of labeled amino acids being greater than 95% was always observed. In cases where only one control/time zero sample was measured, these were for experiments where we started with nonlabeled amino acids. For the studies with inhibitors, equivalent (volume) doses of DMSO were used as controls—inhibitors were solubilized and dosed in DMSO. Between four and twelve time points were used for each experiment. Within each analysis, anywhere from a minimum of three up to sixty EGFR peptide ratios, with an average of about 25 peptide ratios, was used for calculating percent new and old EGFR at each time point.

### 2.1. Preparation of SILAC-Labeled Cells

A431 and H1975 cell lines were purchased from ATCC and were cultured according to ATCC recommendations. PC-9 cells were purchased from RIKEN Cell Bank (Tsukuba, Ibaraki Prefecture, Japan) and were cultured in Gibco RPMI (Life Technologies, Carlsbad, CA, USA) medium with 10% heat-inactivated FBS (Sigma, St. Louis, MO, USA). H3255 cells were from Dr. Bruce E. Johnson at the National Cancer Institute (Bethesda, MD, USA) and were cultured in RPMI, 10% FBS, and ACL-4 supplement (Mediatech Inc., Manassas, VA, USA). PC-9, A431, H1975, and H3255 cells were seeded in 10 cm dishes, one dish with SILAC light medium and one with SILAC heavy medium for each cell line. Heavy arginine [^13^C_6_, ^15^N_4_] and heavy lysine [^13^C_6_, ^15^N_2_] as well as culture media were purchased from Thermo-Fisher. Each cell line was passaged every 3 days (or as required) for a total of 5–8 passages before experiments were initiated in either heavy or light SILAC media (see Thermo Pierce SILAC Protein Quantitation Kits for detailed protocol). At time zero cells were washed at least three times carefully with PBS and new SILAC media were added. In some cases, we started with light labeled cells and other cases with heavy labeled cells. After the defined time the cells were again washed three times with PBS and the cells were scraped and stored at −80°C.

### 2.2. Inhibitor Treatment of EGFR

**Figure 1:**
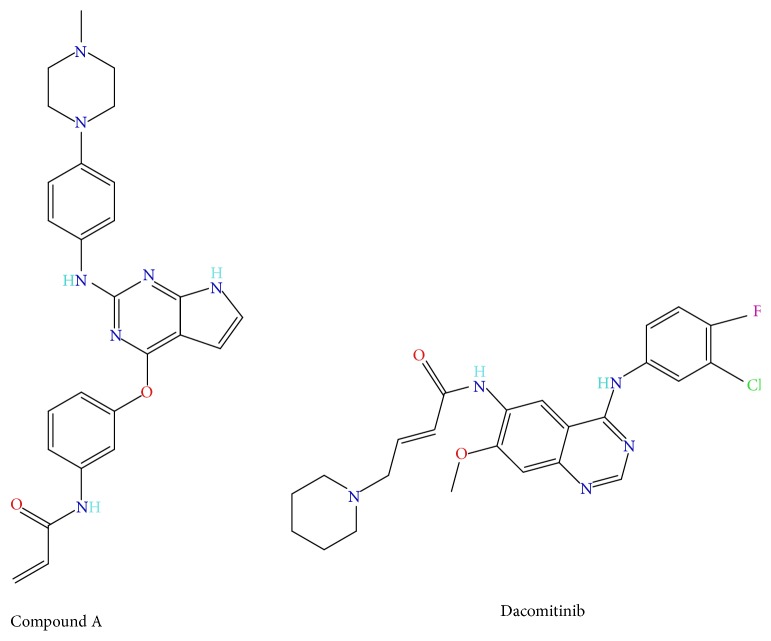
Structures of compound A and dacomitinib, irreversible inhibitors of mutant EGFR. These compounds were dosed in PC-9 cells to test the effects of EGFR irreversible inhibitors on turnover rate of mutant EGFR.

Two irreversible inhibitors of EGFR, dacomitinib, a second-generation compound, and compound A, were used to test effects of irreversible inhibitors on mutant EGFR (Figure 1). SILAC heavy labeled cells were seeded in 100 mm plates 24 hours before inhibitor treatment. Cells were washed twice with PBS and light medium with inhibitor or DMSO. Dacomitinib or compound A at a concentration approximately IC_90_ was added to PC-9 cells and incubated for 0, 0.5, 2, 4, 6, and 24 hours. Cells were washed with PBS at the end of incubation and harvested by scraping. Cells were either processed fresh or stored at −80°C for later analysis.

### 2.3. Enrichment of EGFR and Sample Prep for Mass Spectrometry

All cells were lysed using Cell Signaling Lysis Buffer (Cat. #9803) with brief sonication. The protein concentration was determined by BCA assay (Cat. #23225 of Thermo). EGFR was enriched for mass spec. analysis by immunoprecipitation with EGF receptor (D38B1) XP rabbit mAB (Cat. #5735 of Cell Signaling). Recovered samples were then subjected to trypsin digestion and LC-MS/MS analysis. For the analysis of amino acid recycling, 2-, 4-, 6-, 12-, and 18-hour digestion times with trypsin were tested to determine the optimal time to produce single missed cleavage events. It was found that anywhere between 4 and 18 hours gave the best coverage of the target EGFR protein and the most missed cleavages.

### 2.4. LC-MS/MS Analysis of Peptides

All tryptic digests were analyzed on a Thermo QExactive mass spec. connected to a Thermo Ultimate RSLCnano system. Samples were analyzed using a standard gradient beginning with 0.1% formic acid and 2% acetonitrile and ramped to 40% acetonitrile over 60 minutes at a flow rate of 0.3 *μ*L/min. Thermo EASY-Spray PepMap RSLC C18, 2 *μ*M, 100 Å, 75 *μ*M × 50 cm column was used for the analytical column and the Thermo Acclaim PepMap 100 C18, 3 *μ*M, 100 Å, 75 *μ*M × 2 cm column was used for the loading column. All data was analyzed using Thermo's Proteome Discoverer 1.4 software, searching for each EGFR sequence (WT or mutant) to extract EGFR peptides.

### 2.5. Calculation of Protein Turnover and Amino Acid Recycling

The ratio of labeled peptides was also calculated in Proteome Discoverer 1.4 using a mass precision of 2 ppm to filter labeled and unlabeled peptides. Only peptides with a PEP of less than 0.2 were used for calculations. H/L variance was generally 10–20%. 15–60 EGFR peptides were used for each time point analysis. SILAC ratios were converted to fraction new and old peptides then further analyzed by curve fitting using GraphPad Prism software.

The turnover half-lives were calculated using one-phase exponential decay nonlinear regression where

 fraction old protein at time *t* *P*_*t*_ = *A*_old_/(*A*_old_ + *A*_new_),
 decay rate of protein as *P*_*t*_ approaches 0 with initial labeling from average of all experiments 98.5% *k*_dec_ = −ln⁡(*P*_*t*_/0.985)*t*,
 rate of dilution from cell doubling time is *k*_dil_,
 turnover rate adjusted for cell doubling time *k*_to_ = *k*_dec_ − *k*_dil_,
 turnover half-life *t*_1/2_ = ln⁡(2)/*k*_to_.

For the calculation of amino acid recycling, only peptides with a single missed cleavage were used. The amount of HH (new peptide), HL (new peptide with one recycled amino acid), and LL (old peptide or both amino acids' recycled peptide) was measured. The percent peptide with recycled amino acids was determined by dividing the H/L abundance by the HH + H/L abundance. This was then multiplied by the predicted percent turnover at each time point (calculated from the turnover half-life equation from previous analysis) to get a final percent of total peptides with recycled K or R. Finally, for turnover half-life *t*_1/2_ including recycling effects, the variable *P*_*t*_ was changed as below:

 Recycling modifier *R*_*t*_ = 1 − 100 *∗* (% recycled K and R) at time *t*.
 Fraction old protein at time *tP*_*t*_ = *A*_old_ *∗R*_*t*_/(*A*_old_ *∗R*_*t*_ + *A*_new_).

The new turnover half-life *t*_1/2_ with the effects of recycling included was computed with a constant and variable *R*_*t*_. Although several peptides were observed with two or three missed cleavages, in order to use the data for final percent recycled amino acid, it was necessary to quantify HHL, HLL, and HHH, which were never observed for the same peptide in our sample set.

### 2.6. Calculation of Cellular Doubling Rates

Cells (2000–5000 cells/well depending on the cell line) were seeded in a 96-well microtiter plate. Cell confluence in each well was monitored continuously every 4 hours using an IncuCyte Instrument (IncuCyte ZOOM, ESSEN BioScience, Michigan, USA) until the cell confluence reached 100%. The initial cell seeding number was optimized for each cell line to reach 100% confluency in 3 days. Cell confluence data was averaged from 3 to 10 independent wells and plotted as growth curves (% confluence over time). From a linear portion of the growth curve in the range of 20% to 70% confluency depending on the cell line, a simple extrapolation calculation was conducted to estimate the time required for cell confluency to double.

### 2.7. Cell Viability

Cells (2000–5000 cells/well depending on the cell line) were seeded in a 96-well microtiter plate and allowed to adhere overnight. Compound was added to each well (3-fold serial dilution starting at 10 *μ*M) and incubated for three to five days depending on the cell line. After compound incubation, cell viability was measured utilizing CellTiter-Glo (Promega Corporation, Madison, WI, USA) reagent following the manufacturer's instructions. The resulting luminescence signal was read using an EnVision Multilabel Reader (PerkinElmer, Waltham, MA, USA) plate reader. All assays were run in duplicate and were repeated at least five times.

## 3. Results and Discussion

The half-lives of wild-type and mutant EGFR in biologically relevant cell lines were monitored using SILAC MS. These included lung cancer cell lines H3255 and PC-9 that express the two most commonly found single mutants (L858R, Del19) and H1975 cells that express one of the double mutant forms (L858R/T790M). Wild-type EGFR was assessed in A431 epidermal cancer cells. In order to further refine protein turnover rate measurements, we have investigated the effect of amino acid recycling on protein turnover rates. The effect of inhibitors of mutant EGFR on protein turnover was also examined.

### 3.1. EGFR Turnover in A431 Cells

**Figure 2:**
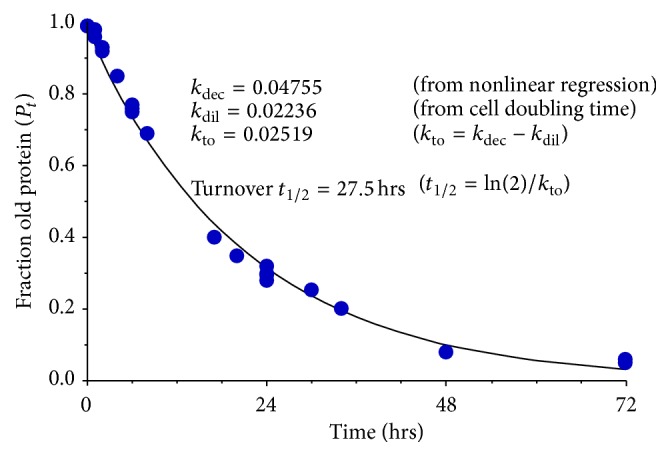
Plot of EGFR turnover rate *t*_1/2_ data from 33 experiments analyzed over a three-month period from A431 cell line. Data was acquired at time points from 0 to 72 hours and incorporation of new K or R was measured. A turnover half-life of 27.5 hours was calculated for WT EGFR.

Our measurements using SILAC MS indicated an EGFR turnover rate *t*_1/2_ of 28 hours in A431 cells without the addition of EGF (Figure 2). The 95% confidence interval for this measurement spans 25.8–29.5 hours. This rate was adjusted for the 31-hour measured cell doubling time. 33 experiments were analyzed varying from measurements at 0 hours to 72 hours after addition of new media. All experiments were done by first labeling proteins with heavy K and R and monitoring changes of heavy to light peptides except for one set of A431 cells where we started with light K and R and then added heavy media. These samples were analyzed at 0, 1, 2, 4, 6, and 8 hours. The L to H and H to L incorporation in EGFR for each set were within 1.5% of each other indicating it did not matter whether we started with light or heavy labeled EGFR. This allowed us to start with light label and chase with heavy label in later experiments in order to save on cost of the heavy media.

### 3.2. EGFR Turnover in Mutant Cell Lines

We investigated several NSCLC cell lines with activating EGFR mutations. PC-9 is a human cell line derived from lung adenocarcinoma cells with EGFR containing the activating mutant deletion 746–750. Cell line H3255 has a single EGFR mutation, L858R, and is very sensitive to tyrosine kinase inhibitors such as gefitinib and erlotinib. H1975, also a NSCLC cell line, contains EGFR with activating mutation L858R and resistance mutation T790M and is resistant to the inhibitor erlotinib. EGFR in H3255 cells had a turnover rate *t*_1/2_ of 10 hours and the PC-9 and H1975 cell lines had turnover rate *t*_1/2_ of 7.2 and 9.2 hours, respectively (Table 1). The cells doubling times ranged from 16 to 42 hours and were used to adjust final turnover time.

The mutations we studied in EGFR cause ligand independent constitutive activation of its kinase activity so the addition of EGF would not significantly affect EGFR activity in the mutant cell lines. As previously reported, addition of EGF to wild-type cells reduced the EGFR half-life from 20 to 9 hours [17]. Not surprisingly, activating mutations did indeed increase protein turnover for EGFR without the addition of EGF. It is suspected that these activating mutations alter the conformation of EGFR, possibly causing more rapid internalization of EGFR and/or allowing greater recognition by the ubiquitin-proteasome pathway and/or lysosomes, thereby increasing the degradation rate.

### 3.3. Effect of Inhibitors on Turnover Rate

The PC-9 turnover rate *t*_1/2_ for the DMSO sample set (7.5 hours) was already a much faster rate when compared to the wild-type A431 cells (28 hours). Since activating EGFR with EGF increases its turnover rate, as do activating mutations, would inhibitors have an additional effect on turnover? To answer this question, we dosed PC-9 cells (Del 746–750 mutant EGFR) with irreversible EGFR inhibitors dacomitinib and the less potent compound A. These inhibitors both covalently bind to Cys797 at the ATP-binding site of mutant EGFR and were dosed near their IC_90_.

Without adjusting for cell doubling time, EGFR turnover rate *t*_1/2_ in PC-9 cells dosed with dacomitinib would be 5.8 hours and dosed with compound A would be 6.9 hours. When PC-9 cells are dosed at or above IC_50_ of dacomitinib, the cells are no longer growing after 72 hours (viability = 1%) and this would suggest that the dilution effect due to cell doubling would be minimized. The EGFR turnover rate *t*_1/2_ would be a maximum of 9.1 hours if cell growth was not affected by dacomitinib, but the actual turnover rate *t*_1/2_ of EGFR is most likely much closer to the unadjusted rate of 5.8 hours. Conversely, compound A treated cells were still 93% viable at 72 hours; thus the turnover rate *t*_1/2_ most likely needs to be adjusted for some amount cell doubling time and could be as high as 12 hours if cell growth rate was unaffected. Due to the complexity of the effects of the small molecules on cellular growth, the turnover rate *t*_1/2_ for EGFR in these experiments was reported as a range (Table 1) based on a minimum of no cell doubling (no cell growth) during the course of the experiment (where *k*_dil_ = 0) to the maximum calculated *k*_dil_ (uninhibited exponential growth).

### 3.4. Recycling of Lysine and Arginine in EGFR

In addition to the LC-MS/MS runs used for calculating protein turnover, we reran several sample sets and incorporated a shorter digestion step to increase the yield of incomplete digestion/missed cleavages. Maximizing missed cleavages gave us more data points for investigating the recycling of the labeled/unlabeled Lys and Arg amino acids in our samples [11, 20]. The cell lines A431, H3255, and H1975 were analyzed for the presence of recycling. All cell lines except for H1975 had peptides where the recycling of amino acids was observed. For the H1975 cell line, eighteen runs were analyzed and there were no peptides identified that had mixed labeled amino acids. This was the case not only for EGFR, but also for all other proteins that were observed in the analysis.

For the A431 cell line, we monitored recycling of K and R in EGFR at 24, 48, and 72 hours starting with light K and R at time 0 and then adding heavy K and R. A total of 6 runs for each time point were used, two biological replicates and 3 technical replicates, with about 40 peptides compared for each time point. For the time points to be deemed valid, we had to observe both the missed cleavage with H/L labeled K or R and the fully labeled peptide.

**Figure 3:**
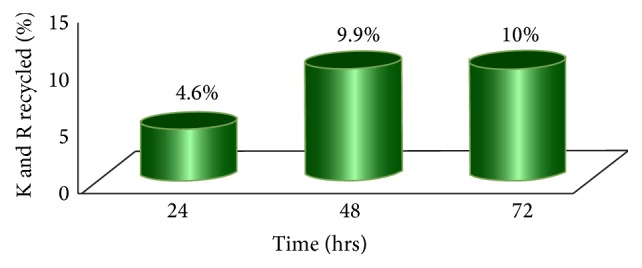
Lysine and arginine recycling in WT EGFR in A431 cells. K and R recycling was calculated by quantifying EGFR peptides with a single missed cleavage from two separate sets of cells, each sample analyzed twice for a total of four MS analyses. The amounts of HH (new peptide), HL (new peptide with recycled amino acid), and LL (old peptide or both amino acids' recycled peptide) were measured. LL was assumed to be composed of only original peptides for this calculation. Recycling appeared to plateau at or before 48 hours which was not unexpected.

At 24 hours 4.6% of the new peptides contained recycled amino acids, and at 48 and 72 hours the new peptides contained 9.9% and 10%, respectively (Figure 3). It is acknowledged that these numbers should be slightly higher because there will be a certain amount of LL peptides where both the L labeled amino acids were recycled. This could not be measured using this technique and was not included in these calculations but the effect would be expected to be very low. The number of recycled LL peptides will increase for a fixed amount of time and then move to zero as the amino acids are broken down by the citric acid cycle instead of moving back into the free amino acid pool. Because the free amino acid pool in cells is limited, it was not surprising that the amount of peptides containing recycled K and R plateaued over the time frame in which we collected data. EGFR turnover in A431 cells would change from 27.5 hours without recycling to 26.0 hours with 5% constant recycling (*R*_*t*_ = 0.95) to 24.4 hours with 10% constant recycling (*R*_*t*_ = 0.90). In this particular case, even the maximum effect of amino acid recycling is not much outside our 95% confidence limits.

**Figure 4:**
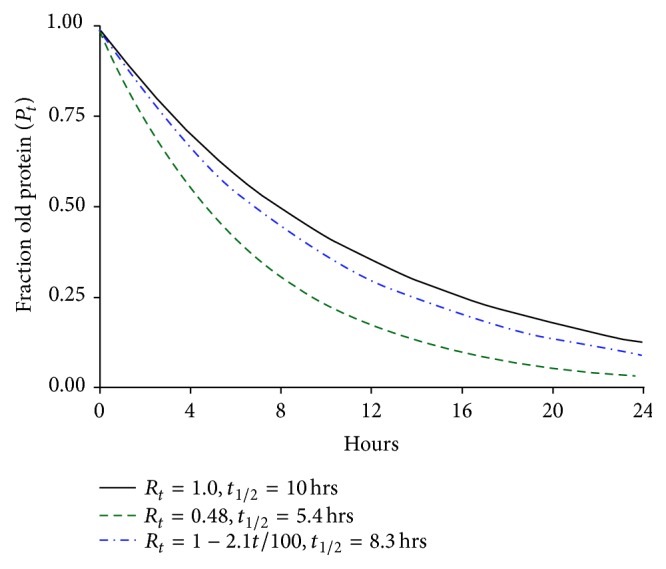
Plot of EGFR turnover *t*_1/2_ data from H3255 cell line showing the effects of no amino acid recycling (*R*_*t*_ = 1.0), at constant 52% (*R*_*t*_ = 0.48), and with a linear increase in recycling up to 24 hours (*R*_*t*_ = 1 − 2.1*t*/100).

The single mutant H3255 cell line with a turnover half-life of 10 hours had 4.8% incorporation of recycled amino acids at 6 hours and 52% at 24 hours. The 4.8% incorporation near its turnover half-life is in line with the A431 cell line recycling. On the other hand, 52% at 24 hours is quite striking. This could be due to the faster turnover rate resulting in increased use of recycled amino acids or different available amino acid pools available in the altered cell line. While this amount of recycling at 24 hours seems quite high, if a linear increase in recycling (variable *R*_*t*_) from 4.8% at 6 hours to 52% at 24 hours is used (*R*_*t*_ = 1 − 2.1*t*/100), then turnover rate *t*_1/2_ is 8.3 hours, not vastly different than the 10 hours calculated without taking recycling into account (Figure 4).

## 4. Conclusions

The study of protein dynamics incorporates many aspects, one of which is protein turnover rate. As proteomic studies have moved from the early days of qualitative assessment of proteins involved in biological pathways to more recent absolute quantitative analysis of changes in pathways due to disease state or other disruptions, the lifetimes of the proteins involved need to be assessed. These insights can allow for a deeper understanding of our targets, which can in turn be used to inform drug discovery strategies aimed at targeting these proteins. Resistance mutations in cancer greatly change the dynamics of more than just protein structure and ligand binding.

We have shown that activating mutations in EGFR increase the overall turnover rate of the protein. The binding of EGF to EGFR activates the kinase and results in internalization of the receptor into the endosomal compartment [27]. Once internalized, EGF receptor still bound to EGF gets degraded while binding to TGF*α* and subsequent dissociation results in recycling of EGFR [28]. It is suspected that these activating mutations enable rapid ubiquitination once internalized as opposed to recycling of EGFR [29]. This in turn leads to increased degradation rates and thereby an increase in turnover of EGFR. Amino acid recycling does affect protein turnover rate measurements and it is understood that rates measured without adjusting for recycling will be slightly high (10–20% for our sample sets). When considering using irreversible inhibitors for cancer treatment, mutations affecting turnover rates can have impact on overall strategy, especially when considering pharmacokinetic properties of lead compounds and dosing.

## Figures and Tables

**Table 1:** Cell lines examined and EGFR turnover rate (*t*_1/2_).

Cell line	Mutation	Cell doubling time (hrs)	Compound	Inhibitor conc. (nM)	Turnover (*t*_1/2_ hrs)
A431	WT	31	N/A		28
H3255	L858R	42	N/A		10
H1975	L858R T790M	23	N/A		9.2
PC-9	Del 746–750	16	DMSO	0	7.5
PC-9	Del 746–750	N/A	Dacomitinib	18	5.8–9.1
PC-9	Del 746–750	N/A	Compound A	360	6.9–12

One wild-type and three mutant EGFR cell lines were analyzed. Cell lines with wild-type EGFR (A431), EGFR with a single activating mutant Del 746–750 (PC-9) or L858R (H3255), and the drug-resistant double mutant L858R/T790M (H1975) EGFR had turnover rate half-lives from 28 to 7.5 hours. These rates were not adjusted for amino acid recycling effects. The EGFR turnover rate half-lives in PC-9 cells dosed with inhibitors compound A and dacomitinib were reported as a range to include impact of cell viability on the measurement.
